# Systematic review and meta‐analysis on prevalence and anamnestic risk factors for erosive tooth wear in the primary dentition

**DOI:** 10.1111/ipd.13250

**Published:** 2024-07-26

**Authors:** Felix Marschner, Philipp Kanzow, Annette Wiegand

**Affiliations:** ^1^ Department of Preventive Dentistry, Periodontology and Cariology University Medical Center Göttingen Göttingen Germany

**Keywords:** erosive tooth wear, meta‐analysis, prevalence, primary dentition, risk factors

## Abstract

**Background:**

Erosive tooth wear is a multifactorial and common condition in children.

**Aim:**

This systematic review and meta‐analysis aimed to determine the prevalence and key risk factors for erosive tooth wear in the primary dentition of children up to 7 years of age.

**Design:**

Electronic databases (PubMed, Embase, Scopus, and Web of Science) were searched in February 2023 for observational studies reporting prevalence and anamnestic risk factors. Additionally, a manual hand search was performed. Meta‐analyses were conducted for the prevalence and odds ratios of identified risk factors. Risk of bias was assessed using the Newcastle–Ottawa scale modified for cross‐sectional studies.

**Results:**

A total of 26 sources, reporting on 23 studies, were included in the systematic review. The overall estimated prevalence of children with erosive tooth wear in the primary dentition amounted to 35.6% (95% CI: 24.8–48.1). Anamnestic factors were structured into domains. Meta‐analyses revealed gastroesophageal reflux disease (GERD; *p*_adj._ = .008; OR = 1.98, 95% CI: 1.37–2.87), consumption of acidic food (*p*_adj._ < .001; OR = 5.14, 95% CI: 3.56–7.42) and acidic drinks (*p*_adj._ < .001; OR = 6.90, 95% CI: 4.64–10.25), holding beverages in the mouth while drinking (*p*_adj._ = .035; OR = 1.82, 95% CI: 1.26–2.63), and snacking regularly (*p*_adj._ = .041; OR = 1.58, 95% CI: 1.18–2.10) to be significantly associated with erosive tooth wear.

**Conclusion:**

Future research should use standardized questionnaires to assess erosive tooth wear and its underlying risk factors (PROSPERO: CRD4202339776).

**Why this paper is important to paediatric dentists**

- GERD, the consumption of acidic food and beverages, holding beverages in the mouth while drinking, and regular snacking were identified as significant risk factors for erosive tooth wear in the primary dentition.
- It is recommended that clinicians increasingly focus on the medical history and potential risk factors to prevent erosive tooth wear in children at an early stage.

## 1 INTRODUCTION

About 40 percent of children below 7 years present erosive tooth wear.^1^ Although a significant proportion of children in the primary dentition is affected by the condition,^2^ erosive tooth wear is not perceived as oral health issue by their parents.^3^ Accordingly, knowledge of potential risk factors is mainly limited to the consumption of acidic beverages,^4^, ^5^ despite the multifactorial etiology of erosive tooth wear. Besides dietary behavior,^6^, ^7^, ^8^ systematic reviews have also identified oral hygiene,^9^ eating disorders,^10^ or reflux^11^, ^12^ as significant risk factors for erosive tooth wear. These systematic reviews, however, specifically focused on the permanent dentition of children, adolescents, and/or adults, rather than on children with primary teeth. As erosive tooth wear in the primary dentition might increase the risk for erosive tooth wear in permanent teeth,^13^ parental knowledge of potential risk factors is crucial for preventing the condition.^14^ So far, only one systematic review and meta‐analysis by Yip et al.^1^ determined potential risk factors for erosive tooth wear in the primary dentition: Only a limited number of risk factors were included in the meta‐analysis, whereas expectable risk factors, such as the consumption of acidic food and beverages or dietary habits, were not statistically analyzed. Hence, this systematic review and meta‐analysis aimed to determine the prevalence of erosive tooth wear in the primary dentition of children up to 7 years of age and to map and analyze potential risk factors for erosive tooth wear.

## 2 MATERIALS AND METHODS

This systematic review was conducted and reported in accordance with the Preferred Reporting Items for Systematic Reviews and Meta‐Analyses (PRISMA) guidelines^15^ and was registered in the PROSPERO database (CRD42023397767) prior to its initiation.

### 2.1 Research question

The study aimed to determine the prevalence of erosive tooth wear and to identify anamnestic risk factors associated with erosive tooth wear in the primary dentition of children up to 7 years of age.

### 2.2 Eligibility criteria

Guided by the PECO (population, exposure, control, and outcome) statement,^16^ eligibility criteria included a population (P) of children up to 7 years of age within the primary dentition. Exposure criteria (E) included children with erosive tooth wear. Control (C) was defined as children without erosive tooth wear, and the outcome (O) included the prevalence and the evaluation of certain risk factors significantly associated with erosive tooth wear. Cross‐sectional observational studies published in English or German were included. Exclusion criteria comprised studies involving populations with permanent dentition, those with insufficient data, case reports, review articles, studies reporting only prevalence and those lacking relevance to anamnestic risk factors.

### 2.3 Search strategy

A systematic literature search was conducted in electronic databases, including MEDLINE via PubMed, Embase via Ovid, Scopus, and Web of Science in February 2023, using the search term (("erosive tooth wear" OR "dental erosive wear" OR "dental erosion") AND (risk OR predictive OR etiological OR predictor OR indicator)). A further hand search using Google and Google Scholar (gray literature) was conducted, and the reference lists of included sources were screened.

### 2.4 Study selection

Following the initial search, duplicative records were identified by reference manager software (EndNote X7). The screening of titles and abstracts was independently conducted by two reviewers (F.M. and P.K.), followed by a full‐text review of potentially eligible sources. Any disagreements between reviewers were resolved through discussion. Reasons for exclusion after the full‐text review are presented in Table S1. In case of missing data (e.g., prevalence of erosive tooth wear and risk factors), authors were contacted via email. After 2 weeks, a second reminder was sent to nonresponders. If authors did not respond or if the required information could not be provided, the studies were excluded from the meta‐analyses.

### 2.5 Data extraction

Data extraction from the included studies was independently performed by two reviewers (F.M. and P.K.) using a pilot‐tested spreadsheet. Extracted information included study characteristics: authors, year of publication, country, continent, number of participants, age, index used for the assessment of erosive tooth wear, prevalence (%), reported risk factors, and results of the statistical analysis (significant vs. nonsignificant risk factors vs. not reported) for each assessed risk factor. Anamnestic risk factors were categorized into domains (sociodemographics, socioeconomics, general health, oral diseases, medication, oral hygiene, food, beverages, dietary habits, and leisure‐related factors). For each reported risk factor, absolute numbers of participants with and without erosive tooth wear were extracted to facilitate quantitative synthesis.

### 2.6 Data synthesis and meta‐analysis

Meta‐analysis was performed for the prevalence (%) of erosive tooth wear. Studies assessing erosive tooth wear using different indices were entered as subgroups. Additionally, multivariable meta‐regression analysis was performed to investigate the relationship between the prevalence of erosive tooth wear and the effect of the kind of index, the examination mode (full‐mouth recording vs. reference teeth), geographic location of study (continents), and the year of publication.

Identified risk factors and their respective significance in each study were visually presented in a heatmap. Additionally, meta‐analyses of odds ratios (ORs) were conducted for each risk factor domain. Within each domain, individual risk factors, which were uniformly reported as dichotomized variables, were entered as subgroups, and pooled effect estimates were derived for each subgroup.

All statistical analyses were performed using the software R (version 4.3.1, www.r‐project.org) using the packages “meta” (version 7.0‐0) and “metafor” (version 4.4‐0). The level of significance was set to α = .05. As multiple meta‐analyses on potential risk factors were performed and multiple subgroups were assessed, resulting p‐values were adjusted for multiple testing according to Bonferroni–Holm. Statistical heterogeneity was assessed using Cochran's *Q* and *I* ^2^ statistics.^17^ Heterogeneity was considered if *p*‐values from Cochran's *Q* were <.05 or in cases of *I* ^*2*^ values ≥50% (i.e., indicating moderate or high heterogeneity).^18^ Due to high heterogeneity, random effects meta‐analyses were performed throughout the study. To validate the results of the random effects model, a sensitivity analysis was conducted in which the random effects model was compared with the fixed effects model. Publication bias was assessed visually by examining funnel plots and conducting Egger's regression intercept tests.^19^

### 2.7 Quality assessment

Quality and risk of bias of the included sources were assessed using the modified Newcastle–Ottawa scale for cross‐sectional studies (maximum 10 stars).^20^ The evaluation was independently conducted by two reviewers (F.M. and P.K.). All sources were rated with stars (*) and categorized into three components (selection: maximum 5 stars, comparability: maximum 2 stars, and outcome: maximum 3 stars). High quality was indicated by 9–10 stars, good quality by 7–8 stars, satisfactory quality by 5–6 stars, and unsatisfactory quality by 0–4 stars.^20^, ^21^ Further details are provided in Table S2.

### 2.8 Evidence assessment

The Grading of Recommendations Assessment, Development, and Evaluation (GRADE) system was employed to appraise the quality of evidence.^22^ Evaluation criteria included the risk of bias, inconsistency, indirectness, imprecision, and publication bias. These criteria served as indicators for the up‐ or downgrading of the evidence for the findings.

## 3 RESULTS

### 3.1 Literature search

**FIGURE 1:**
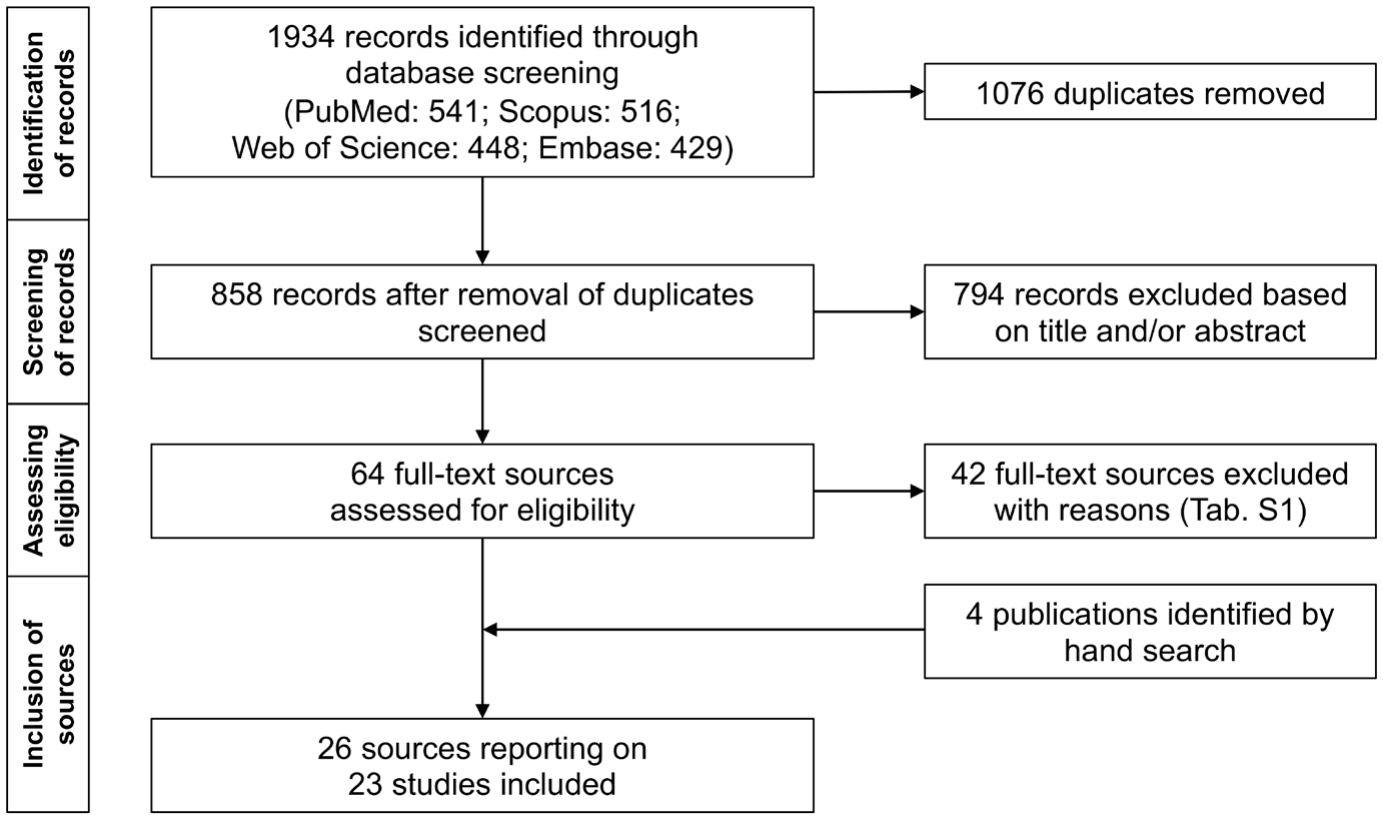
Flowchart of literature search and study selection.

A total of 1934 potentially relevant records were identified through electronic databases. Following the removal of duplicates and the exclusion of records based on title and/or abstract, 64 sources were considered eligible for full‐text examination. After a full‐text reading, additional 42 sources were excluded, and the details are outlined in Table S1. Four publications were included through manual search. A total of 26 sources^3^, ^23^, ^24^, ^25^, ^26^, ^27^, ^28^, ^29^, ^30^, ^31^, ^32^, ^33^, ^34^, ^35^, ^36^, ^37^, ^38^, ^39^, ^40^, ^41^, ^42^, ^43^, ^44^, ^45^, ^46^, ^47^ reporting on 23 studies were included in this systematic review. The PRISMA flowchart of the literature search is shown in Figure 1.

### 3.2 Study characteristics

The study characteristics are reported in Table 1. A total of 16 777 participants (range: 100–1993 participants), with an age range of 2–7 years and originating from different continents, were examined in 23 observational studies. Regarding the distribution per continent, 11 studies were conducted in Asia,^25^, ^26^, ^27^, ^29^, ^30^, ^34^, ^35^, ^37^, ^40^, ^41^, ^43^, ^44^, ^45^ seven in Europe,^3^, ^23^, ^28^, ^33^, ^39^, ^42^, ^46^, ^47^ four in South America,^24^, ^32^, ^36^, ^38^ and one in Oceania.^31^

**TABLE 1:** Characteristics of included studies.

No.	Study and country	Number of participants (% males)	Age range (year)	Recruitment	Index used	Index application
1	Millward et al. 1994, United Kingdom	178 (44.9)	4–5	Schools	TWI	Palatal, labial, and occlusal surfaces of all primary teeth
2	Al‐Majed et al. 2002, Saudi Arabia	354 (100)	5–6	Elementary schools	O'Brien Index	Palatal and labial surfaces of the primary maxillary incisors and molars
3	Al‐Malik et al. 2002a & Al‐Malik et al. 2002b, Saudi Arabia	987 (NR)	2–5	Schools	O'Brien Index	Primary maxillary incisors
4	Harding et al. 2003, Ireland	202 (52.0)	5	Schools	O'Brien Index	Palatal and labial surfaces of primary maxillary incisors
5	Deshpande and Hugar et al. 2004, India	100 (60.0)	5–6	Schools	mod. TWI	All primary teeth
6	Luo et al. 2005, China	1949 (52.7)	3–5	Kindergartens	O'Brien Index	Primary maxillary incisors
7	Wiegand et al. 2006, Germany	463 (53.1)	2–7	Kindergartens	O'Sullivan Index	All primary teeth
8	Rios et al. 2007, Brazil	356 (NR)	6	Schools	TWI	All primary teeth
9	Nayak et al. 2010 & Nayak et al. 2012, India	1002 (56.1)	5	Schools	mod. TWI	All primary teeth
10	Murakami et al. 2011, Brazil	967 (47.9)	3–4	National Children's Vaccination day	O'Brien Index	All primary teeth
11	Raza and Hashim et al. 2012, United Arab Emirates	207 (46.4)	5–6	Schools	mod. TWI	Buccal, lingual, and incisal/occlusal surfaces of the primary molars and canines
12	Mantonanaki et al. 2013, Greece	605 (50.6) (examination only) 524 (NR) (examination and questionnaire)	5	Kindergartens	BEWE	All primary teeth
13	Moimaz et al. 2013, Brazil	1993 (49.4)	4–6	Preschool	TWI	All primary teeth
14	Huang et al. 2015, Australia	154 (44.8)	2–4	Community health clinics	mod. TWI	All primary teeth
15	Tao et al. 2015, China	1837 (51.6)	3–6	Kindergartens	O'Sullivan Index	All primary teeth
16	Gopinath 2016, United Arab Emirates	403 (48.1)	5	Kindergartens	O'Brien Index	Palatal surfaces of the primary maxillary incisors
17	Tschammler et al. 2016, Germany	775 (52.3)	3–6	Kindergartens	BEWE	All primary teeth
18	Al‐Dlaigan et al. 2017, Saudi Arabia	388 (47.4)	3–5	Kindergartens	O'Brien Index	All primary teeth
19	Duangthip et al. 2018, China	1204 (44.9)	3–5	Kindergartens	BEWE	All primary teeth
20	Gatt and Attard 2019 & Gatt and Attard 2022, Malta	775 (53.3)	3–5	Schools	BEWE	All primary teeth
21	Maharani et al. 2019b, Indonesia	691 (53.5)	5	Kindergartens	BEWE	All primary teeth
22	Pereira et al. 2020, Brazil	888 (51.4)	5	Preschools	O'Brien Index	All primary teeth
23	Tvilde et al. 2021, Norway	380 (48.0)	5	Public dental service clinics	SEPRS	Buccal and palatal surfaces of the primary maxillary incisors and canines as well as occlusal surfaces of primary molars

Abbreviations: BEWE, Basic Erosive Wear Examination; mod. TWI, modified Tooth Wear Index; NR, not reported; SEPRS, Simplified Erosion Partial Recording System; TWI, Tooth Wear Index.

Erosive tooth wear was measured using various indices, most studies utilized the O'Brien Index and O'Brien‐modified indices (34.78%).^24^, ^27^, ^29^, ^36^, ^40^, ^42^, ^43^, ^44^, ^45^ Additionally, the Tooth Wear Index (TWI) and TWI‐modified indices were applied in 30.43% of the studies.^31^, ^32^, ^34^, ^35^, ^37^, ^38^, ^41^, ^46^ The Basic Erosive Wear Examination was used in five studies^3^, ^25^, ^26^, ^28^, ^33^, ^47^ to measure erosive tooth wear, whereas the remaining studies used the O'Sullivan Index^30^, ^39^ and the Simplified Erosion Partial Recording System (SEPRS).^23^

**FIGURE 2:**
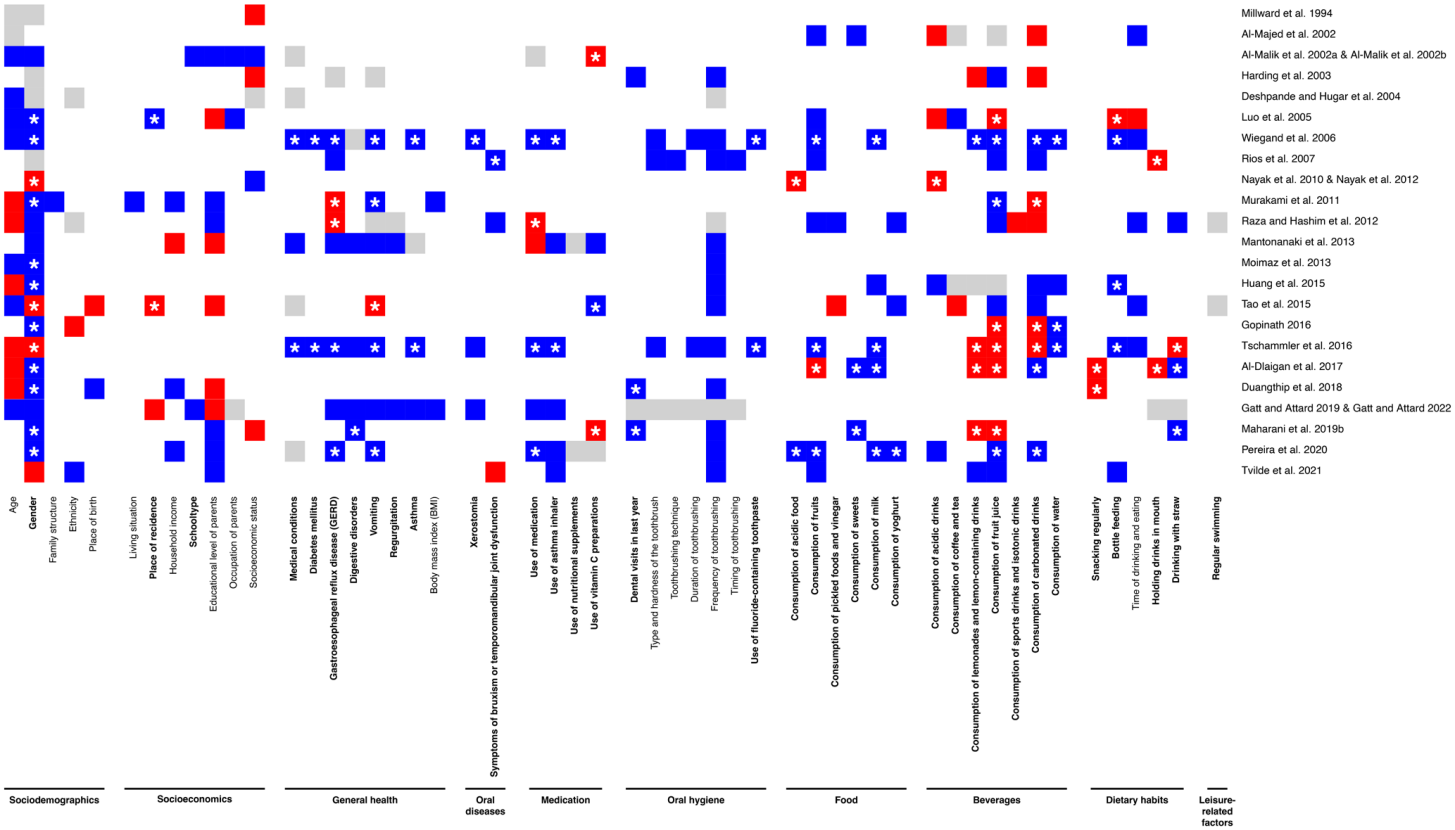
Heatmap presenting the investigated risk factors for each study. Horizontal axis of the heatmap represent the various risk factors, whereas the vertical axis represent the studies included in chronological order. Red square: Risk factor was significant with respect to erosive tooth wear in the respective study; blue square: Risk factor was not significant with respect to erosive tooth wear in the respective study; gray square: Not specified in the respective study whether the risk factor was significant with respect to erosive tooth wear or not; * Data included in meta‐analyses; in bold print, risk factors included in meta‐analyses.

The reported anamnestic risk factors in the studies varied but could be structured into the following domains: sociodemographics, socioeconomics, general health, oral diseases, medication, oral hygiene, food, beverages, dietary habits, and leisure‐related factors. Figure 2 displays the reported anamnestic risk factors for each individual study.

### 3.3 Quality assessment of included sources

Using the modified Newcastle–Ottawa scale for cross‐sectional studies, the risk of bias of each included source was evaluated. Most of the studies received a good quality score.^27^, ^28^, ^29^, ^30^, ^31^, ^32^, ^34^, ^35^, ^37^, ^38^, ^40^, ^41^, ^42^, ^44^ Eleven sources were rated to have high quality^3^, ^23^, ^24^, ^25^, ^26^, ^33^, ^36^, ^39^, ^43^, ^45^, ^47^ and only one source had satisfactory quality.^46^ Detailed results of the risk of bias are reported in Table S2.

### 3.4 Prevalence of erosive tooth wear

**FIGURE 3:**
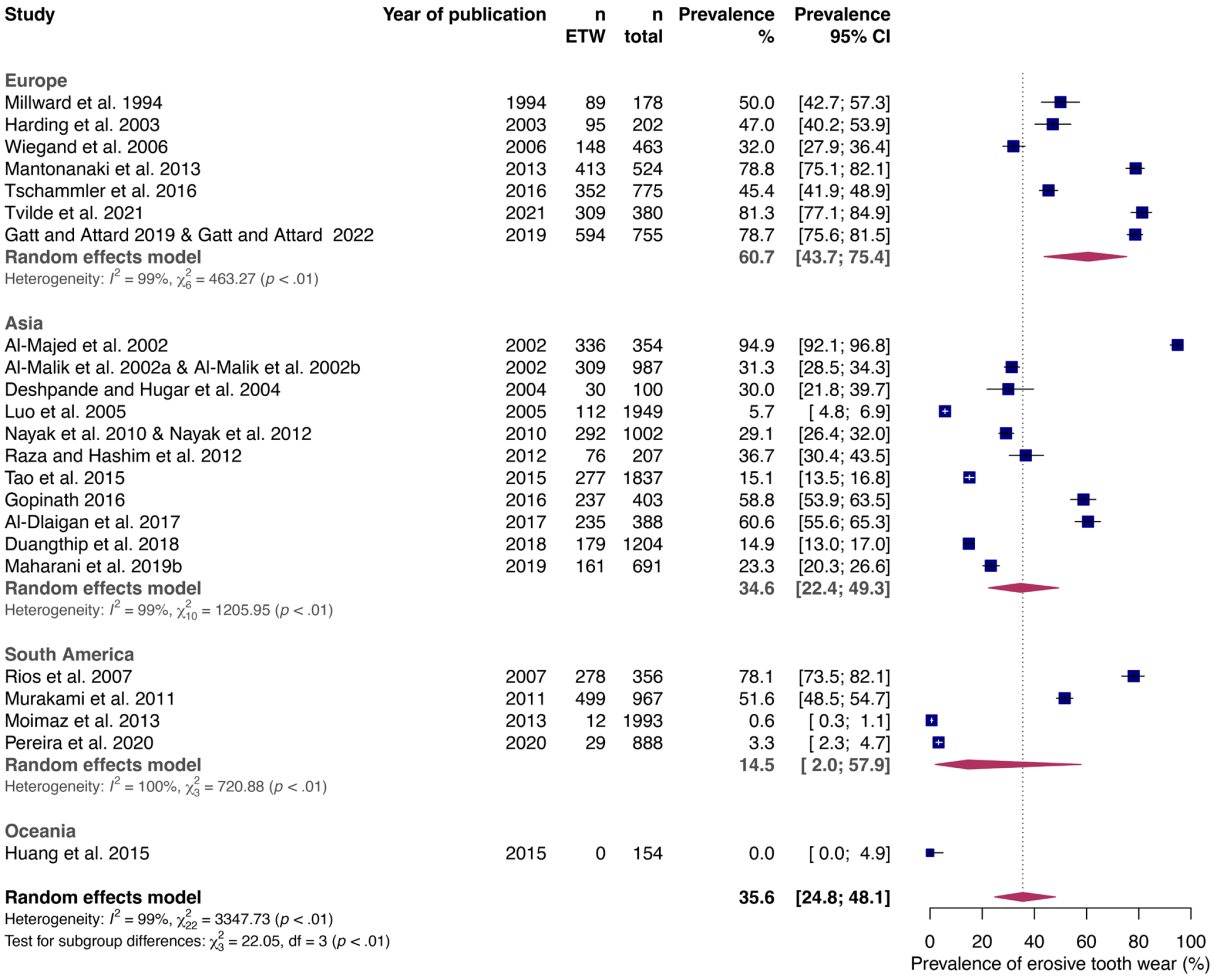
Forest plot showing the pooled random effects estimate of erosive tooth wear prevalence (% and 95% confidence intervals) in the primary dentition up to 7 years of age. Studies from different continents are shown as subgroups with their pooled effect estimates. CI, confidence interval; ETW, erosive tooth wear.

All studies included in this systematic review were considered in the meta‐analysis of the prevalence of erosive tooth wear.^3^, ^23^, ^24^, ^25^, ^26^, ^27^, ^28^, ^29^, ^30^, ^31^, ^32^, ^33^, ^34^, ^35^, ^36^, ^37^, ^38^, ^39^, ^40^, ^41^, ^42^, ^43^, ^44^, ^45^, ^46^, ^47^ The overall estimated prevalence of children with erosive tooth wear in the primary dentition up to 7 years of age amounted to 35.6% (95% CI: 24.8–48.1, Figure 3.) Statistical and graphical analyses did not indicate relevant publication bias (Figure S1).

Meta‐regression showed a significant impact of the continent and the number of participants per study, whereas the used indices and the publication year did not significantly impacted the prevalence of erosive tooth wear (Table 2).

**TABLE 2:** Meta‐regression analyses regarding the impact of different variables on the logit of the prevalence of erosive tooth wear (logit %).

Variable	Estimate	95% CI	*p*‐Value
Year of publication	−0.064	−0.191 to 0.062	.320
Index used (ref.: BEWE)			
mod. TWI	−2.399	−4.958 to 0.160	.066
O'Brien Index	−1.182	−3.624 to 1.259	.343
O'Sullivan Index	−0.812	−3.008 to 1.385	.469
SEPRS	1.016	−2.373 to 4.405	.557
TWI	−1.863	−5.036 to 1.309	.250
Only some teeth examined (ref.: all)	0.144	−1.644 to 1.932	.874
Continent (ref.: Europe)			
Asia	0.627	−1.078 to 2.333	.471
Oceania	**−4.536**	**−8.937 to −0.134**	**.043**
South America	0.416	−2.327 to 3.158	.766
Number of participants	**−0.002**	**−0.003 to −0.001**	**<.001**

Abbreviations: BEWE, Basic Erosive Wear Examination; CI, confidence interval; mod. TWI, modified Tooth Wear Index; SEPRS, Simplified Erosion Partial Recording System; TWI, Tooth Wear Index; in bold print, statistical significance (*p*‐value < .05).

### 3.5 Anamnestic risk factors for erosive tooth wear

Fifty‐two anamnestic risk factors and their potential association with erosive tooth wear were documented in the included studies (Figure 2). The mapping of risk factors revealed a substantial variability in the assessment of anamnestic risk factors. Sociodemographic factors, such as age (69.57% of the studies)^3^, ^26^, ^27^, ^28^, ^30^, ^31^, ^32^, ^34^, ^36^, ^39^, ^40^, ^41^, ^43^, ^44^, ^45^, ^46^, ^47^ and gender (95.65% of the studies),^3^, ^23^, ^24^, ^25^, ^26^, ^27^, ^28^, ^29^, ^30^, ^31^, ^32^, ^33^, ^34^, ^35^, ^36^, ^37^, ^38^, ^39^, ^40^, ^41^, ^42^, ^43^, ^45^, ^46^, ^47^ were most often investigated. Additionally, general health conditions and the consumption of various beverages were common risk factors, though their evaluation showed variability and was not consistently performed across studies.

In total, nine meta‐analyses were conducted, and 29 risk factors were entered as subgroups of the respective domains (data regarding leisure‐related factors were not comparable in two groups). The studies included in the meta‐analyses are depicted in Figure 2. The ORs with their 95% confidence intervals (CI) for each included risk factors as well as the pooled effect estimates in the meta‐analyses and summary of findings are listed in Table 3. The presence of gastroesophageal reflux disease (GERD) (*p*_adj._ = .008; OR = 1.98, 95% CI: 1.37–2.87), consumption of acidic food (*p*_adj._ < .001; OR = 5.14, 95% CI: 3.56–7.42), consumption of acidic drinks (*p*_adj._ < .001; OR = 6.90, 95% CI: 4.64–10.25), holding beverages in the mouth while drinking (*p*_adj._ = .035; OR = 1.82, 95% CI: 1.26–2.63), and snacking regularly (*p*_adj._ = .041; OR = 1.58, 95% CI: 1.18–2.10) were significantly associated with the presence of erosive tooth wear. A protective association was found for the risk factors drinking with a straw (*p*_adj._ = .019; OR = 0.58, 95% CI: 0.42–0.80) and consumption of fruits (*p*_adj._ = .002; OR = 0.66, 95% CI: 0.54–0.81). None of the other risk factors exhibited a significant association with erosive tooth wear (*p*_adj._ ≥ .05). Detailed results and forest plots for each meta‐analysis are presented in Figures S2–S10. Regarding publication bias, there was only one domain (general health) with a serious risk, and funnel plots and results of Egger's regression intercept tests are shown in Figure S11. The results of the conducted sensitivity analysis (random effects vs. fixed effects models) are shown in Table S3, confirming the results of the random effects model. Additionally, in the fixed effects model, a statistically significant association between consumption of fruit juice (*p*_adj._ < .001; OR = 1.44, 95% CI: 1.26–1.66) and consumption of water (*p*_adj._ < .001; OR = 0.20, 95% CI: 0.16–0.26) with the presence of erosive tooth wear was found, with high heterogeneity (*I* ^2^ = 88%, *p* < .01 and *I* ^2^ = 97%, *p* < .01).

**TABLE 3:** Anamnestic risk factors, results of meta‐analyses and summary of findings.

Anamnestic risk factors (studies included in meta‐analysis)	Included in meta‐analysis	Summary of findings						
		Study event rates (%)		Relative effect			Anticipated absolute effects	
		Without	With	*p*‐Value	*p*_adj._‐Value	OR (95% CI)	Risk without	Risk with
*Sociodemographics*								
Age	–^a^	–	–	–	–	–	–	–
Gender (13)	Male vs. female	5146/10211 (50.4)	1304/2490 (52.4)	.115	>.999	1.12 (0.97–1.29)	504 per 1000	28 more per 1000 (8 fewer to 63 more)
Family structure	–^a^	–	–	–	–	–	–	–
Ethnicity	–^a^	–	–	–	–	–	–	–
Place of birth	–^a^	–	–	–	–	–	–	–
*Socioeconomics*								
Living situation	–^b^	–	–	–	–	–	–	–
Place of residence (2)	Rural (suburban) vs. urban	1789/3397 (52.7)	220/389 (56.6)	.969	>.999	1.01 (0.60–1.71)	527 per 1000	2 more per 1000 (126 fewer to 129 more)
Household income	–^a^	–	–	–	–	–	–	–
Schooltype	–^b^	–	–	–	–	–	–	–
Educational level of parents	–^a^	–	–	–	–	–	–	–
Occupation of parents	–^a^	–	–	–	–	–	–	–
Socioeconomic status	–^a^	–	–	–	–	–	–	–
*General health*								
Medical conditions (2)	Yes vs. no	28/738 (3.8)	33/500 (6.6)	.097	>.999	1.56 (0.92–2.62)	38 per 1000	20 more per 1000 (3 fewer to 56 more)
Diabetes mellitus (2)	Yes vs. no	0/738 (0)	1/500 (0.2)	.432	>.999	3.61 (0.15–89.00)	0 per 1000	0 more per 1000 (0 fewer to 0 more)
Gastroesophageal reflux disease (5)	Yes vs. no	164/2194 (7.5)	75/1104 (6.8)	**<.001**	**.008**	**1.98 (1.37–2.87)**	75 per 1000	63 more per 1000 (25 to 114 more)
Digestive disorders (1)	Yes vs. no	65/530 (12.3)	21/161 (13.0)	.793	>.999	1.07 (0.63–1.82)	123 per 1000	7 more per 1000 (42 fewer to 80 more)
Vomiting (5)	Yes vs. no	876/3567 (24.6)	198/1299 (15.2)	.007	.139	1.36 (1.09–1.70)	246 per 1000	61 more per 1000 (16 to 111 more)
Regurgitation	–^b^	–	–	–	–	–	–	–
Asthma (2)	Yes vs. no	23/724 (3.2)	23/500 (4.6)	.454	>.999	1.26 (0.69–2.28)	32 per 1000	8 more per 1000 (10 fewer to 38 more)
Body mass index	–^a^	–	–	–	–	–	–	–
*Oral diseases*								
Xerostomia (1)	Yes vs. no	0/315 (0)	0/148 (0)	NA	NA	NA	NA	NA
Symptoms of bruxism or temporo‐mandibular joint dysfunction (1)	Yes vs. no	12/51 (23.5)	84/268 (31.3)	.267	>.999	1.48 (0.74–2.98)	235 per 1000	78 more per 1000 (50 fewer to 243 more)
*Medication*								
Use of medication (4)	Yes vs. no	199/1708 (11.7)	33/605 (5.5)	.066	>.999	1.56 (0.97–2.52)	117 per 1000	54 more per 1000 (3 fewer to 133 more)
Use of asthma inhaler (2)	Yes vs. no	19/718 (2.6)	15/500 (3.0)	.992	>.999	1.00 (0.50–2.00)	26 per 1000	0 fewer per 1000 (13 fewer to 25 more)
Use of nutritional supplements	–^b^	–	–	–	–	–	–	–
Use of vitamin C preparations (3)	Yes vs. no	590/2606 (22.6)	151/727 (20.8)	.033	.665	1.88 (1.05–3.35)	226 per 1000	128 more per 1000 (9 to 268 more)
*Oral hygiene*								
Dental visits in last year (2)	Yes vs. no	354/1543 (22.9)	81/339 (23.9)	.730	>.999	0.95 (0.72–1.26)	229 per 1000	9 fewer per 1000 (53 fewer to 43 more)
Type and hardness of toothbrush	–^a^	–	–	–	–	–	–	–
Toothbrushing technique	–^a^	–	–	–	–	–	–	–
Duration of toothbrushing	–^a^	–	–	–	–	–	–	–
Frequency of toothbrushing	–^a^	–	–	–	–	–	–	–
Timing of toothbrushing	–^a^	–	–	–	–	–	–	–
Use of fluoride‐containing toothpaste (2)	Yes vs. no	628/679 (92.5)	437/474 (92.2)	.708	>.999	1.09 (0.70–1.70)	925 per 1000	6 more per 1000 (29 fewer to 29 more)
*Food*								
Consumption of acidic food (2)	Yes vs. no	**973/1596 (61.0)**	**257/294 (87.4)**	**<.001**	**<.001**	**5.14 (3.56–7.42)**	**610 per 1000**	**279 more per 1000** **(238 to 311 more)**
Consumption of fruits (4)	Yes vs. no	**1196/1715 (69.7)**	**354/742 (47.7)**	**<.001**	**.002**	**0.66 (0.54–0.81)**	**697 per 1000**	**94 fewer per 1000** **(46 to 143 fewer)**
Consumption of pickled foods and vinegar	–^b^	–	–	–	–	–	–	–
Consumption of sweets (2)	Yes vs. no	548/683 (80.2)	222/396 (56.1)	.763	>.999	0.92 (0.52–1.61)	802 per 1000	14 fewer per 1000 (124 fewer to 65 more)
Consumption of milk (4)	Yes vs. no	1272/1732 (73.4)	515/757 (68.1)	.251	>.999	0.79 (0.52–1.18)	734 per 1000	48 fewer per 1000 (145 fewer to 31 more)
Consumption of yoghurt (1)	Yes vs. no	559/858 (65.2)	23/29 (79.3)	.120	>.999	2.06 (0.83–5.11)	652 per 1000	142 more per 1000 (43 fewer to 254 more)
*Beverages*								
Consumption of acidic drinks (1)	Yes vs. no	**384/710 (54.1)**	**260/292 (89.0)**	**<.001**	**<.001**	**6.90 (4.64–10.25)**	**541 per 1000**	**350 more per 1000 (304 to 383 more)**
Consumption of coffee and tea	–^b^	–	–	–	–	–	–	–
Consumption of lemonades and lemon‐containing drinks (4)	Yes vs. no	247/1392 (17.7)	159/876 (18.2)	.960	>.999	1.03 0.35–2.99	177 per 1000	4 more per 1000 (107 fewer to 214 more)
Consumption of fruit juice (8)	Yes vs. no	1842/4412 (41.7)	888/1732 (51.3)	.143	>.999	1.69 (0.84–3.43)	417 per 1000	130 more per 1000 (42 fewer to 293 more)
Consumption of sports drinks and isotonic drinks	–^b^	–	–	–	–	–	–	–
Consumption of carbonated drinks (6)	Yes vs. no	1107/2353 (47.0)	389/1480 (26.3)	.138	>.999	1.64 (0.85–3.16)	470 per 1000	123 more per 1000 (40 fewer to 267 more)
Consumption of water (3)	Yes vs. no	800/885 (90.4)	414/722 (57.3)	.093	>.999	0.10 (0.01–1.47)	904 per 1000	419 fewer per 1000 (818 fewer to 29 more)
*Dietary habits*								
Snacking regularly (2)	Yes vs. no	**554/1166 (47.5)**	**309/414 (74.6)**	**.002**	**.041**	**1.58 (1.18–2.10)**	**475 per 1000**	**113 more per 1000 (41 to 180 more)**
Time of drinking and eating	–^a^	–	–	–	–	–	–	–
Holding drinks in mouth (2)	Yes vs. no	**61/206 (29.6)**	**151/343 (44.0)**	**.002**	**.035**	**1.82 (1.26–2.63)**	**296 per 1000**	**138 more per 1000 (50 to 229 more)**
Bottle feeding (4)	Yes vs. no	846/2339 (36.2)	397/626 (63.4)	.136	>.999	1.30 (0.92–1.84)	362 per 1000	62 more per 1000 (19 fewer to 149 more)
Drinking with straw (3)	Yes vs. no	**819/1026 (79.8)**	**392/622 (63.0)**	**<.001**	**.019**	**0.58 (0.42–0.80)**	**798 per 1000**	**102 fewer per 1000 (38 to 174 fewer)**
*Leisure‐related risk factors*								
Regular swimming	–^b^	–	–	–	–	–	–	–

Abbreviations: CI, confidence interval; NA, not applicable; OR, odds ratio.

^a^ Data not comparable in two groups.

^b^ Lack of data.

In bold print, statistical significance (*p*_adj._‐value < .05).

### 3.6 Assessment of evidence

The quality of evidence related to the prevalence of children with erosive tooth wear in the primary dentition up to 7 years of age and domains of anamnestic risk factors is presented in Table 4. According to GRADE recommendations, the overall evidence has been downgraded to a low or very low level due to the reliance on observational studies.

**TABLE 4:** Assessment of the evidence of prevalence of erosive tooth wear and risk factors according to the grading of recommendations assessment, development, and evaluation system.

Outcome (studies)	Number of participants	Risk of bias	Inconsistency	Indirectness	Imprecision	Publication bias	Overall certainty of evidence
Prevalence of erosive tooth wear (23)	16 757	Not serious	Serious	Not serious	Not serious	Not serious	⨁◯◯◯ Very low
*Domain*							
Sociodemographics (13)	12 701	Not serious	Serious	Not serious	Serious	Not serious	⨁◯◯◯ Very low
Socioeconomics (2)	3786	Not serious	Serious	Not serious	Serious	Not serious	⨁◯◯◯ Very low
General health (7)	5764	Not serious	Not serious	Not serious	Not serious	Serious	⨁◯◯◯ Very low
Oral diseases (2)	782	Not serious	NA	Not serious	Serious	Not serious	⨁◯◯◯ Very low
Medication (7)	5439	Not serious	Serious	Not serious	Not serious	Not serious	⨁◯◯◯ Very low
Oral hygiene (4)	3035	Not serious	Not serious	Not serious	Not serious	Not serious	⨁⨁◯◯ Low
Food (6)	4150	Not serious	Serious	Not serious	Serious	Not serious	⨁◯◯◯ Very low
Beverages (9)	7146	Not serious	Serious	Not serious	Serious	Not serious	⨁◯◯◯ Very low
Dietary habits (8)	5397	Not serious	Serious	Not serious	Serious	Not serious	⨁◯◯◯ Very low
Leisure‐related risk factors (0)	0	NA	NA	NA	NA	NA	NA

Abbreviation: NA, not applicable.

## 4 DISCUSSION

The overall estimated prevalence of erosive tooth wear in the primary dentition amounted to 35.6%, which is slightly lower than the prevalence of erosive tooth wear in children below 7 years of age, which was reported in a recent meta‐analysis (39.6%).^1^ Only population‐based studies that reported the prevalence of erosive tooth wear and potential anamnestic risk factors (assessed via questionnaires) were included, whereas studies on children presenting specific conditions (e.g., only healthy children) or without assessing anamnestic risk factors were excluded. As methodological conditions might affect the prevalence of erosive tooth wear,^48^ meta‐regression analysis was performed to consider the effect of the kind of index, the examination mode (full‐mouth recording vs. reference teeth), geographic location of study (continents), and year of publication. Neither the kind of index nor the examination mode affected the overall prevalence of erosive tooth wear, increasing the overall validity of the analysis. With regard to the geographic location, no effect could be observed except for the continent Australia.^31^ Nevertheless, as the dataset behind is extracted from only one study reporting a prevalence of zero percent, this effect should not be overestimated. Interestingly, the meta‐regression analysis revealed no effect of the year of publication, which might reflect an overall increase/decrease in erosive tooth wear over the past decades. Although few studies reported an increase in the prevalence of erosive tooth wear over time^3^, ^14^, ^28^ this observation could not be confirmed by our analysis. Also, the number of participants per study impacted the prevalence of erosive tooth wear with larger studies reporting a lower prevalence. Future studies should employ an adequate sample size, and individual results from smaller studies should be interpreted with caution.

Mapping of risk factors demonstrated a high variability of the assessed anamnestic risk factors and the need for standardized questionnaires. Structuring the risk factors in different domains showed that sociodemographic factors, namely gender and age, general health conditions, and consumption of different kinds of beverages, were most often, but not consistently, assessed. Besides, the mapping revealed no factor to be consistently reported to be significantly associated with erosive tooth wear, making the identification of the most relevant risk factors impossible. Therefore, meta‐analyses of anamnestic factors in the different domains were performed. We found GERD, the consumption of acidic food and beverages, holding beverages in the mouth while drinking, and snacking regularly to be significantly associated with erosive tooth wear in the primary dentition. These results are in accordance with meta‐analyses on potential risk factors in the permanent dentition, also demonstrating that GERD^11^ or the consumption of carbonated drinks, natural acidic fruit juices, confectionery, and snacks^6^ increased the risk for erosive tooth wear. An interesting finding revealed a protective association with the consumption of fruits. Regarding fruit consumption, this can be attributed to the inability to precisely distinguish between the types of fruits (whether acidic or not). Previous research found that the consumption of fruits (i.e., nonacidic fruits) other than apples or citrus may decrease the risk for erosive tooth wear.^49^ Moreover, the timing of dietary acid intake might be important, as fruit intake between meals, but not with meals, might increase the risk for erosive tooth wear.^50^ Our finding that the use of a straw is likely to have a protective effect on erosive tooth wear is explained by the fact that direct contact with the tooth surface is avoided, as confirmed in a previous study.^51^

The main limitation of the meta‐analyses of risk factors, however, is that only dichotomous factors could be included, whereas several other variables (e.g., age and frequency of toothbrushing) could not be used as they were not reported in a standardized manner, and raw data were not provided by the authors to allow for further analysis (e.g., meta‐regression analysis). Consequently, specific domains, such as socioeconomics and oral hygiene are underrepresented in the meta‐analyses. Moreover, the number of studies per subgroup (i.e., risk factor) was small in many cases, limiting the overall validity of our results. The availability of raw data would have allowed for additional studies being entered into the meta‐analyses. Finally, heterogeneity was found to be high in most meta‐analyses. Hence, random effects models were applied throughout the study. Overall, the certainty of the evidence must be regarded as low or very low due to the study design of included studies (i.e., observational studies) and the abovementioned limitations.

The limitations underlie the need for standardization of assessment and reporting of anamnestic risk factors in children, ideally with raw data included as supplemental material. The strength of this systematic review and meta‐analysis, however, aimed to include all assessed risk factors rather than to focus on a single specific risk factor.^52^ The methodological strengths of this review included the systematic literature search across four different databases, the additional search of gray literature, and the use of GRADE to assess the evidence for each outcome. Additionally, it can be noted that this review is one of the first to provide an overview of nearly all potential anamnestic risk factors associated with erosive tooth wear and graphically represent them. This innovative approach could contribute significantly to advancing research in the field and aiding in the prevention of erosive tooth wear in primary dentition.

This systematic review and meta‐analysis highlights the high prevalence of erosive tooth wear in primary dentition and identifies significant anamnestic risk factors. The findings underscore the importance of standardized assessment methods and comprehensive data reporting to better understand and prevent this condition. Paediatric dentists should be aware of the significant risk factors, such as GERD and the consumption of acidic foods and beverages, to implement more effective preventive measures.

## AUTHOR CONTRIBUTIONS

A.W. conceived the ideas; P.K. and F.M. collected the data; P.K. analyzed the data; and F.M. and A.W. led the writing.

## FUNDING INFORMATION

This research did not receive any specific grant from funding agencies in the public, commercial, or not‐for‐profit sectors.

## CONFLICT OF INTEREST STATEMENT

The authors have no conflict of interest to declare.

## Supporting information


Appendix S1

## Data Availability

All data analyzed during this study are included in this article or online in Appendix S1. Further inquiries can be directed to the corresponding author.
