# Supplementary material for: Systematic review and meta‐analysis on prevalence and anamnestic risk factors for erosive tooth wear in the primary dentition
Source: Int J Paediatr Dent. 2024 Jul 26;35(2):389–404. doi: 10.1111/ipd.13250 (PMC11788517; doi:10.1111/ipd.13250)
Supplement: Supplementary file 1 — Appendix S1 [file IPD-35-389-s001.docx]

**Appendices**

Supplemental Table S1. Full-text sources excluded with reasons.

| **Reason for exclusion**  **(studies excluded)** | **Studies** |
| --- | --- |
| Language  (1) | Rihter et al. 2015 |
| Case-control-study  (5) | Brandt et al. 2017, Jastaniyah et al. 2019, Pantelewicz & Olczak-Kowalczyk 2020, Rezende et al. 2019, Tong et al. 2014 |
| Review  (1) | Ahmed & Donovan 2015 |
| Conference paper  (1) | Ellis et al. 2022 |
| Only prevalence or incidence reported  (3) | Affshana 2015, Ganss et al. 2001, Murakami et al. 2016 |
| No clinical examination  (1) | Xhonga-Oja & Valdmanis 1986 |
| Only diagnostic topic  (1) | Mitchell et al. 2003 |
| Irrelevant topic  (5) | Anderson et al. 2020, Gatou and Mamai Homata 2012, Johansson 2002, Nihtyanova et al. 2018¸Taji et al. 2010 |
| Age  (4) | Alaraudanjoki et al. 2016, Chuajedong et al. 2002, Pedrão et al. 2018, Waterhouse et al. 2008 |
| Permanent and mixed dentition  (18) | Ayers et al. 2002, Al-Ashtal et al. 2017, Dantham et al. 2021, de Andrade et al. 2016, El Aidi et al. 2011, Fung & Messer 2013, Garduno-Picazo et al. 2020, Hasselkvist & Arnrup 2021, Huew et al. 2012, Huew et al. 2011, Karki et al. 2021, Liu et al. 2021, Ludovichetti et al. 2022, Mangueira et al. 2009, Margaritis et al. 2011, Marqués Martínez et al. 2019, Nahás Pires Corrêa et al. 2011, Sales-Peres et al. 2013 |
| Patient with special needs or high risk for erosive tooth wear  (2) | Mohamed et al. 2021, Tschammler et al. 2019 |

Supplemental Table S2. Quality and risk of bias of the included sources.

|  | Selection | | | |  | Comparability |  | Outcome | |  | Total score |  | Quality |
| --- | --- | --- | --- | --- | --- | --- | --- | --- | --- | --- | --- | --- | --- |
|  | 1. | 2. | 3. | 4. |  | 5. |  | 6. | 7. |  |  |  |  |
| Millward et al. 1994 | * | * |  | ** |  | * |  | * |  |  | ****** |  | satisfactory |
| Al-Majed et al. 2002 |  | * |  | ** |  | ** |  | * | * |  | ******* |  | good |
| Al-Malik et al. 2002a | * | * | * | ** |  | ** |  | * | * |  | ********* |  | high |
| Al-Malik et al. 2002b | * | * | * | ** |  | ** |  | * | * |  | ********* |  | high |
| Harding et al. 2003 | * | * |  | ** |  | ** |  | * | * |  | ******** |  | good |
| Deshpande and Hugar et al. 2004 | * | * |  | ** |  | ** |  | * |  |  | ******* |  | good |
| Luo et al. 2005 | * | * |  | ** |  | ** |  | * | * |  | ******** |  | good |
| Wiegand et al. 2006 | * | * | * | ** |  | ** |  | * | * |  | ********* |  | high |
| Rios et al. 2007 | * | * |  | ** |  | ** |  | * | * |  | ******** |  | good |
| Nayak et al. 2010 | * | * |  | ** |  | ** |  | * | * |  | ******** |  | good |
| Murakami et al. 2011 | * | * | * | ** |  | ** |  | * | * |  | ********* |  | high |
| Nayak et al. 2012 | * | * |  | ** |  | ** |  | * | * |  | ******** |  | good |
| Raza and Hashim et al. 2012 | * | * |  | ** |  | ** |  | * | * |  | ******** |  | good |
| Mantonanaki et al. 2013 | * | * | * | ** |  | ** |  | * | * |  | ********* |  | high |
| Moimaz et al. 2013 | * | * |  | ** |  | ** |  | * | * |  | ******** |  | good |
| Huang et al. 2015 | * | * |  | ** |  | ** |  | * | * |  | ******** |  | good |
| Tao et al. 2015 | * | * |  | ** |  | ** |  | * | * |  | ******** |  | good |
| Gopinath 2016 | * | * |  | ** |  | ** |  | * | * |  | ******** |  | good |
| Tschammler et al. 2016 | * | * |  | ** |  | ** |  | * | * |  | ******** |  | good |
| Al-Dlaigan et al. 2017 | * | * |  | ** |  | ** |  | * | * |  | ******** |  | good |
| Duangthip et al. 2018 | * | * | * | ** |  | ** |  | * | * |  | ********* |  | high |
| Gatt and Attard 2019 | * | * | * | ** |  | ** |  | * | * |  | ********* |  | high |
| Maharani et al. 2019b | * | * | * | ** |  | ** |  | * | * |  | ********* |  | high |
| Pereira et al. 2020 | * | * | * | ** |  | ** |  | * | * |  | ********* |  | high |
| Tvilde et al. 2021 | * | * | * | ** |  | ** |  | * | * |  | ********* |  | high |
| Gatt and Attard 2022 | * | * | * | ** |  | ** |  | * | * |  | ********* |  | high |

1. Representativeness of the sample (max. *); 2. Sample size (max. *); 3. Non-responds (max. *); 4. Ascertainment of exposure (max. **); 5. The subjects in different outcome groups are comparable, based on the study design or analysis. Confounding factors are controlled. (max. **); 6. Assessment of outcome (max. **); 7. Statistical test (max. *)

Supplemental Table S3. Sensitivity analysis (random effects vs. fixed effects models).

| **Outcome / risk factor** | | **Outcome measure** | **Random effects model** | | **Fixed effects model** | |
| --- | --- | --- | --- | --- | --- | --- |
| Prevalence of erosive tooth wear | | % | 35.6 (24.8-48.1) | – | 37.3 (36.3-38.2) | – |
| Socio-demographics | Gender | OR | 1.12 (0.97-1.29) | p_adj._>0.999 | 1.12 (1.02-1.24) | p_adj._=0.338 |
| Socio-economics | Place of residence | OR | 1.01 (0.60-1.71) | p_adj._>0.999 | 1.13 (0.91-1.40) | p_adj._>0.999 |
| General health | Medical conditions | OR | 1.56 (0.92-2.62) | p_adj._>0.999 | 1.56 (0.92-2.62 | p_adj._>0.999 |
|  | Diabetes mellitus | OR | 3.61 (0.15-89.0) | p_adj._>0.999 | 3.61 (0.15-89.0) | p_adj._>0.999 |
|  | Gastroesophageal reflux disease | OR | **1.98 (1.37-2.87)** | **p_adj._=0.008** | **1.99 (1.37-2.88)** | **p_adj._=0.006** |
|  | Vomiting | OR | 1.36 (1.09-1.70) | p_adj._=0.139 | 1.36 (1.09-1.70) | p_adj._=0.112 |
|  | Asthma | OR | 1.26 (0.69-2.28) | p_adj._>0.999 | 1.26 (0.69-2.28) | p_adj._>0.999 |
|  | Digestive disorders | OR | 1.07 (0.63-1.82) | p_adj._>0.999 | 1.07 (0.63-1.82) | p_adj._>0.999 |
| Oral diseases | Xerostomia | OR | – | – | – | – |
|  | Symptoms of Bruxism or Temporomandibular  joint dysfunction | OR | 1.48 (0.74-2.98) | p_adj._>0.999 | 1.48 (0.74-2.98) | p_adj._>0.999 |
| Medication | Use of vitamin C preparations | OR | 1.88 (1.05-3.35) | p_adj._=0.665 | 1.37 (1.09-1.71) | p_adj._=0.107 |
|  | Use of medication | OR | 1.56 (0.97-2.52) | p_adj._>0.999 | 1.56 (0.97-2.51) | p_adj._=0.900 |
|  | Use of asthma inhaler | OR | 1.00 (0.50-2.00) | p_adj._>0.999 | 1.00 (0.50-2.01) | p_adj._>0.999 |
| Oral hygiene | Use of fluoride-containing tooth paste | OR | 1.09 (0.70-1.70) | p_adj._>0.999 | 1.09 (0.70-1.70) | p_adj._>0.999 |
|  | Dental visits in last year | OR | 0.95 (0.72-1.26) | p_adj._>0.999 | 0.95 (0.72-1.26) | p_adj._>0.999 |
| Food | Consumption of fruits | OR | **0.66 (0.54-0.81)** | **p_adj._=0.002** | **0.67 (0.55-0.82)** | **p_adj._=0.003** |
|  | Consumption of milk | OR | 0.79 (0.52-1.18) | p_adj._>0.999 | 0.75 (0.57-0.97) | p_adj._=0.475 |
|  | Consumption of acidic food | OR | **5.14 (3.56-7.42)** | **p_adj._<0.001** | **5.15 (3.56-7.43)** | **p_adj._<0.001** |
|  | Consumption of sweets | OR | 0.92 (0.52-1.61) | p_adj._>0.999 | 1.02 (0.69-1.50) | p_adj._>0.999 |
|  | Consumption of yoghurt | OR | 2.06 (0.83-5.11) | p_adj._>0.999 | 2.06 (0.83-5.11) | p_adj._>0.999 |
| Beverages | Consumption of fruit juice | OR | 1.69 (0.84-3.43) | p_adj._>0.999 | **1.44 (1.26-1.66)** | **p_adj._<0.001** |
|  | Consumption of lemonades and lemon-containing drinks | OR | 1.03 (0.35-2.99) | p_adj._>0.999 | 1.16 (0.92-1.47) | p_adj._>0.999 |
|  | Consumption of carbonated drinks | OR | 1.64 (0.85-3.16) | p_adj._>0.999 | 1.31 (1.09-1.57) | p_adj._=0.063 |
|  | Consumption of water | OR | 0.10 (0.01-1.47) | p_adj._>0.999 | **0.20 (0.16-0.26)** | **p_adj._<0.001** |
|  | Consumption of acidic drinks | OR | **6.90 (4.64-10.25)** | **p_adj._<0.001** | **6.90 (4.64-10.25)** | **p_adj._<0.001** |
| Dietary habits | Bottle feeding | OR | 1.30 (0.92-1.84) | p_adj._>0.999 | 1.19 (0.96-1.48) | p_adj._>0.999 |
|  | Holding drinks in mouth | OR | **1.82 (1.26-2.63)** | **p_adj._=0.035** | **1.82 (1.26-2.63)** | **p_adj._=0.031** |
|  | Drinking with straw | OR | **0.58 (0.42-0.80)** | **p_adj._=0.019** | **0.56 (0.43-0.72)** | **p_adj._<0.001** |
|  | Snacking regularly | OR | **1.58 (1.18-2.10)** | **p_adj._=0.041** | **1.58 (1.18-2.10)** | **p_adj._=0.036** |

OR, Odds ratio; CI, confidence interval; **in bold print**, statistical significance p_adj._-value<0.05


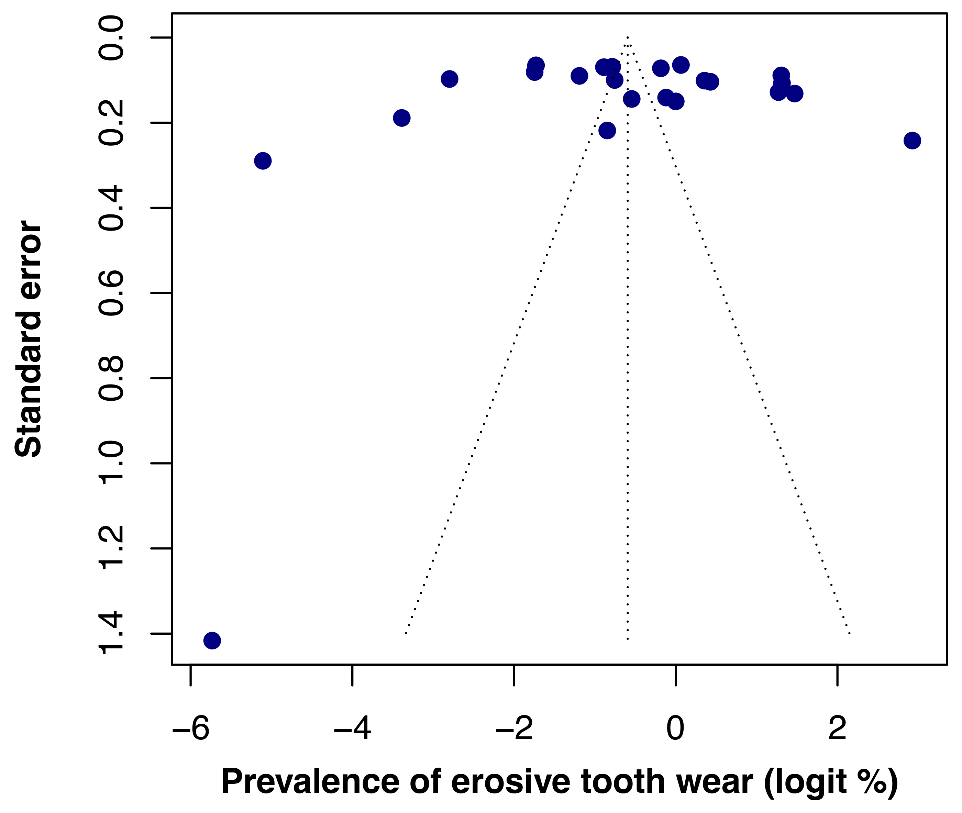


Supplemental Figure 1. Funnel plot plotting standard errors against the logit of erosive tooth wear prevalence (logit %). Egger’s regression intercept test does not indicate publication bias (p=0.792).


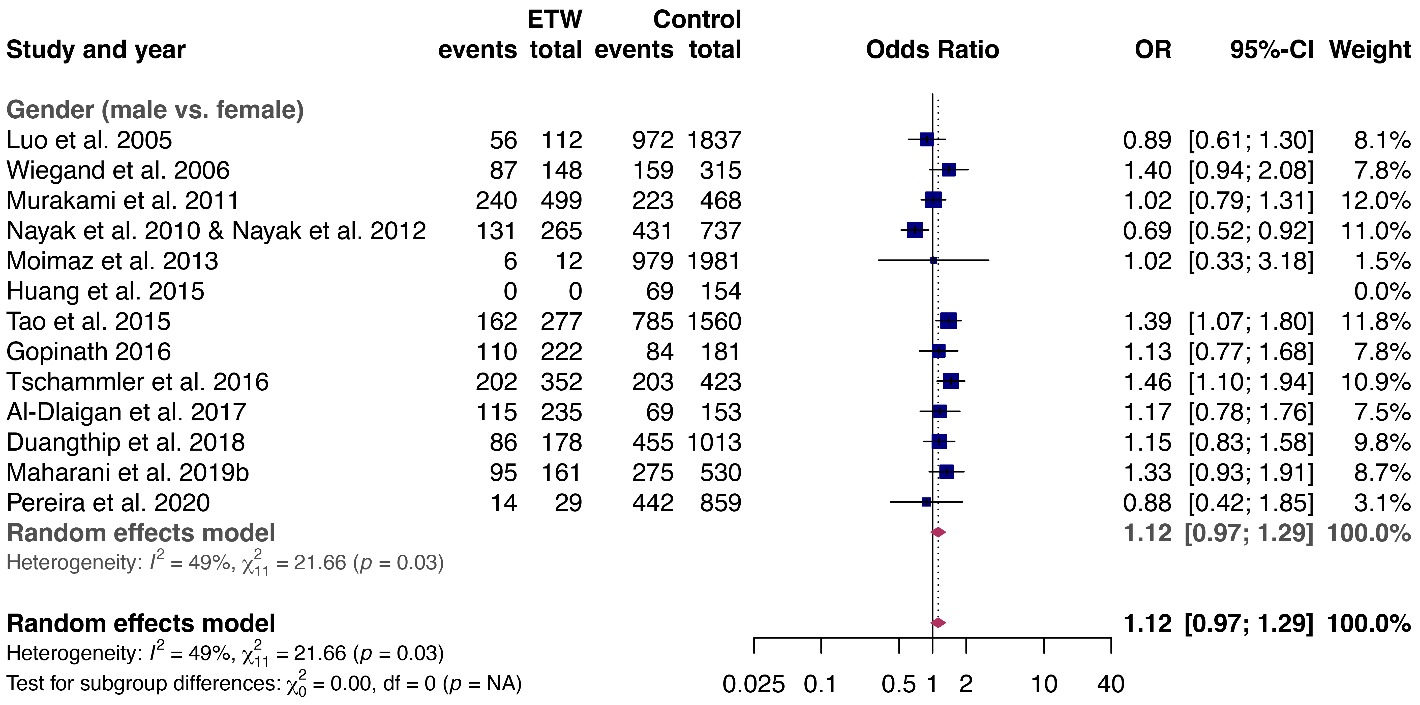


Supplemental Figure 2. Forest plot for potential socio-demographic risk factors showing the association of male gender (ref.: female) and the presence of erosive tooth wear in the primary dentition up to 7 years of age. Odds ratios, 95% confidence intervals, and pooled random-effect estimates (diamonds) are shown. ETW, erosive tooth wear; OR, odds ratio; CI, confidence interval.


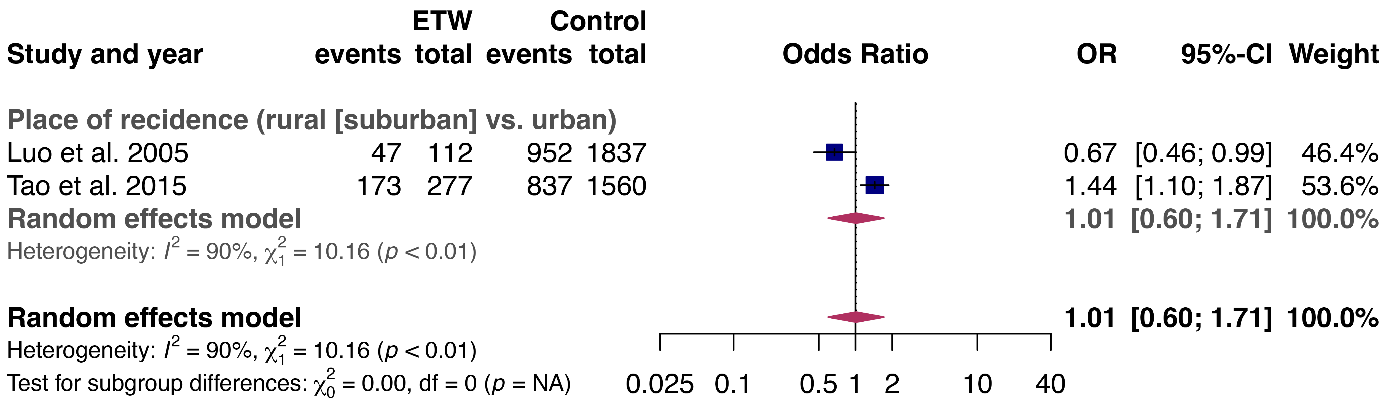


Supplemental Figure 3. Forest plot for potential socio-demographic risk factors showing the association of rural or suburban place of residence (ref.: urban) and the presence of erosive tooth wear in the primary dentition up to 7 years of age. Odds ratios, 95% confidence intervals, and pooled random-effect estimates (diamonds) are shown. ETW, erosive tooth wear; OR, odds ratio; CI, confidence interval.


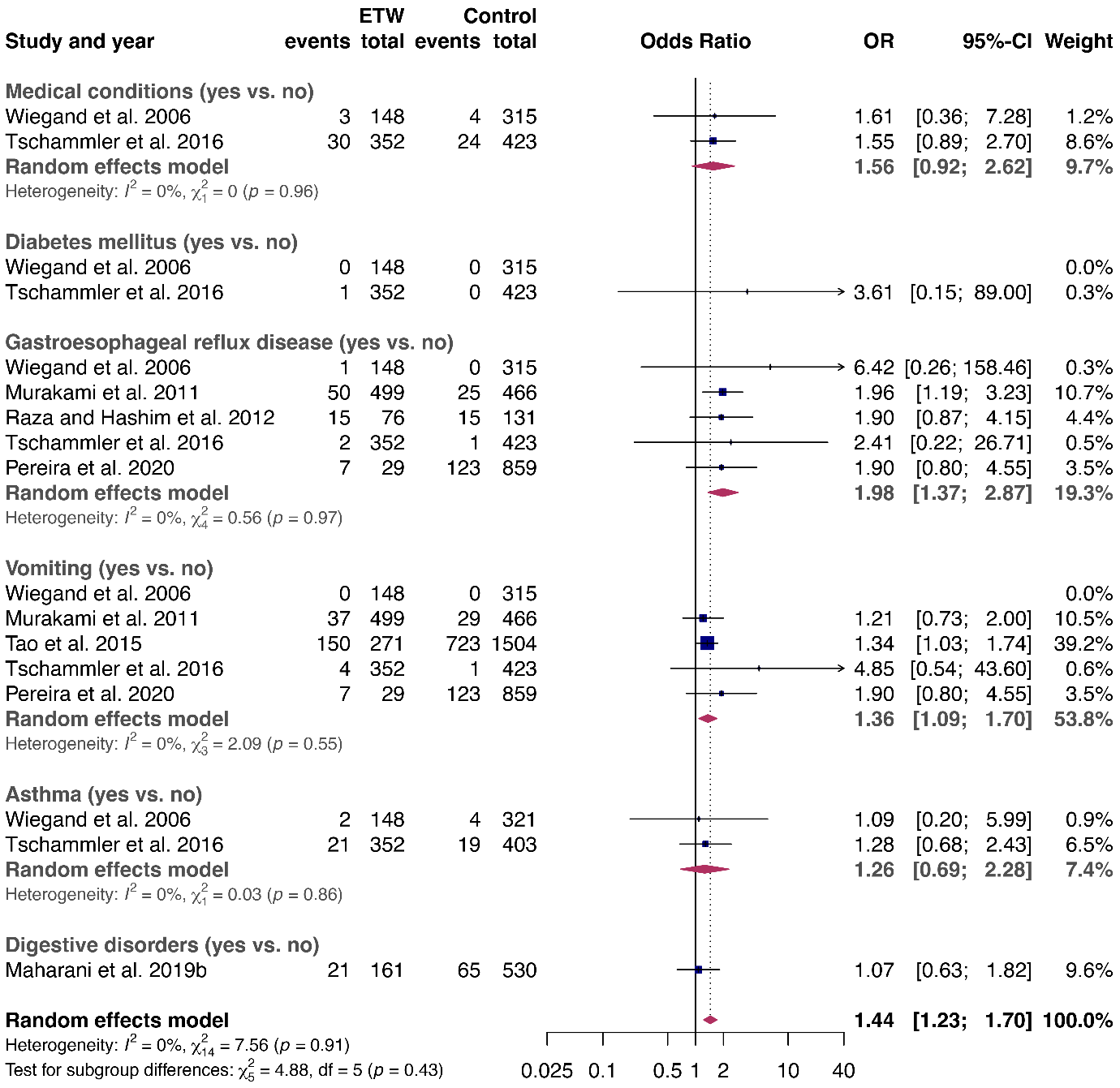


Supplemental Figure 4. Forest plot showing the association of potential general health risk factors and the presence of erosive tooth wear in the primary dentition up to 7 years of age. Odds ratios, 95% confidence intervals, and pooled random-effect estimates (diamonds) are shown. ETW, erosive tooth wear; OR, odds ratio; CI, confidence interval.


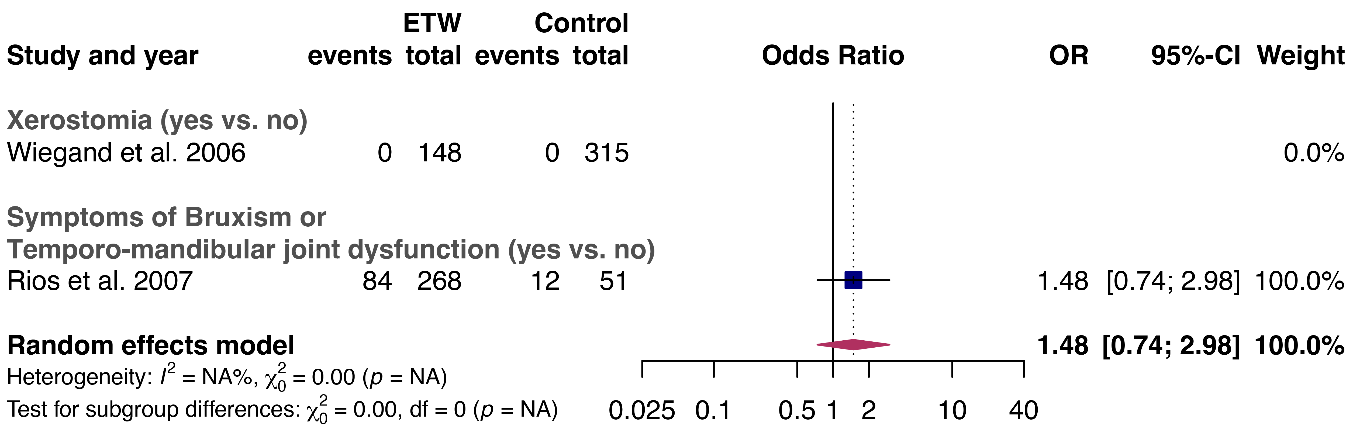


Supplemental Figure 5. Forest plot showing the association of potential risk factors related to oral diseases and the presence of erosive tooth wear in the primary dentition up to 7 years of age. Odds ratios, 95% confidence intervals, and pooled random-effect estimates (diamonds) are shown. ETW, erosive tooth wear; OR, odds ratio; CI, confidence interval.


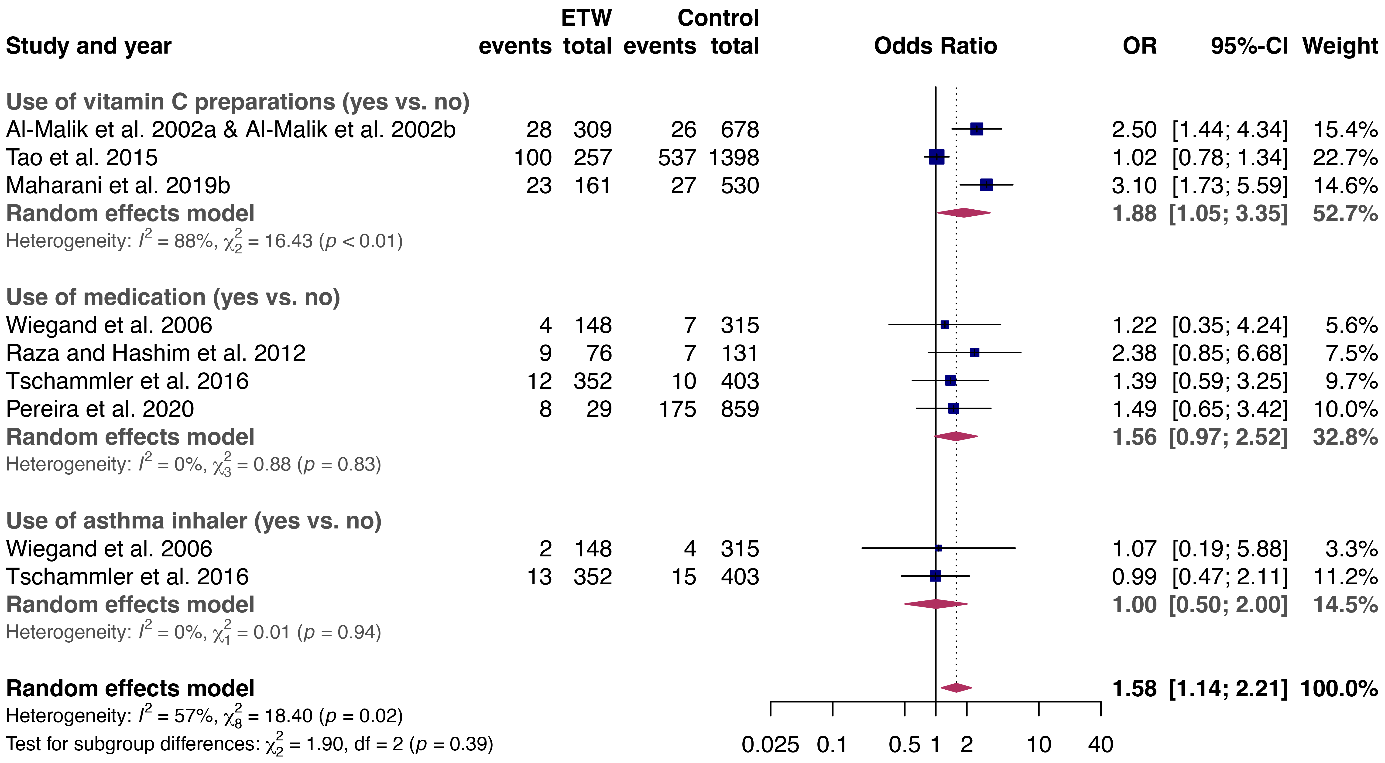


Supplemental Figure 6. Forest plot showing the association of potential risk factors related to medication and the presence of erosive tooth wear in the primary dentition up to 7 years of age. Odds ratios, 95% confidence intervals, and pooled random-effect estimates (diamonds) are shown. ETW, erosive tooth wear; OR, odds ratio; CI, confidence interval.


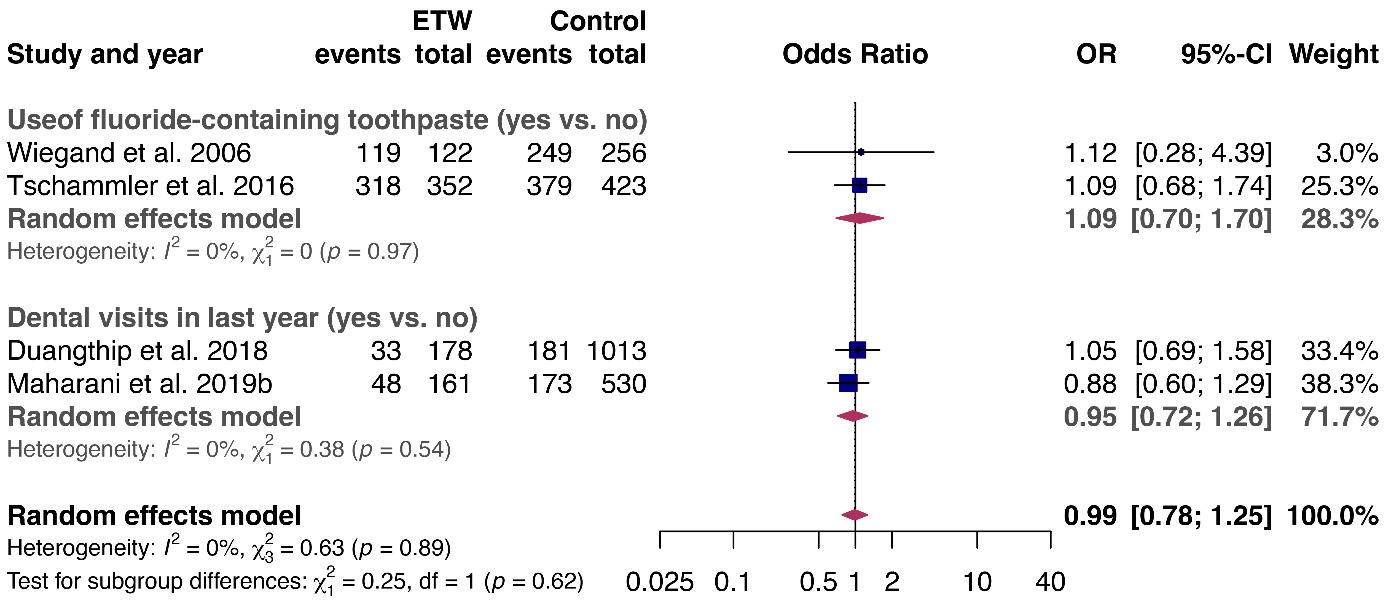


Supplemental Figure 7. Forest plot showing the association of potential risk factors related to oral hygiene and the presence of erosive tooth wear in the primary dentition up to 7 years of age. Odds ratios, 95% confidence intervals, and pooled random-effect estimates (diamonds) are shown. ETW, erosive tooth wear; OR, odds ratio; CI, confidence interval.


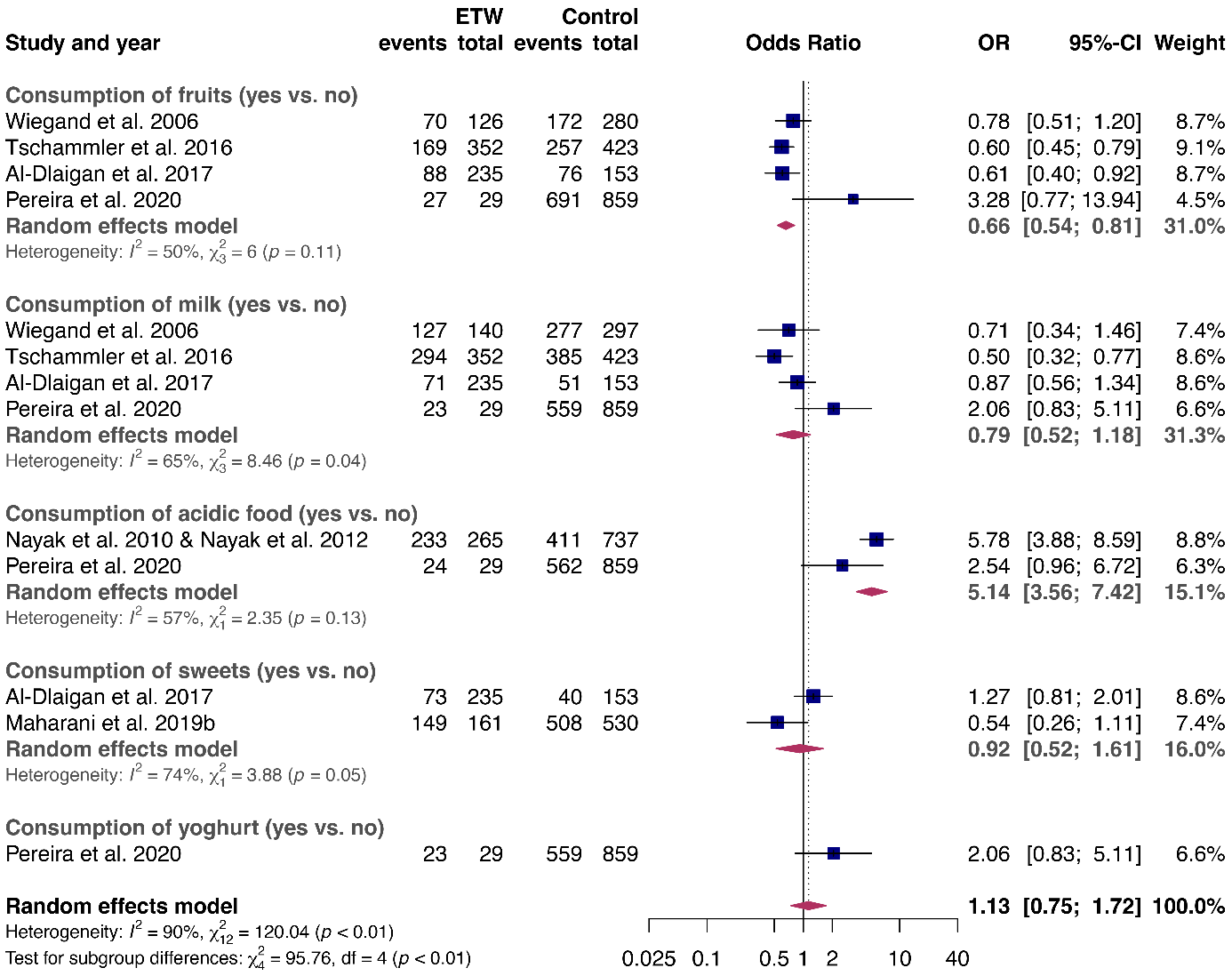


Supplemental Figure 8. Forest plot showing the association of potential risk factors related to food consumption and the presence of erosive tooth wear in the primary dentition up to 7 years of age. Odds ratios, 95% confidence intervals, and pooled random-effect estimates (diamonds) are shown. ETW, erosive tooth wear; OR, odds ratio; CI, confidence interval.


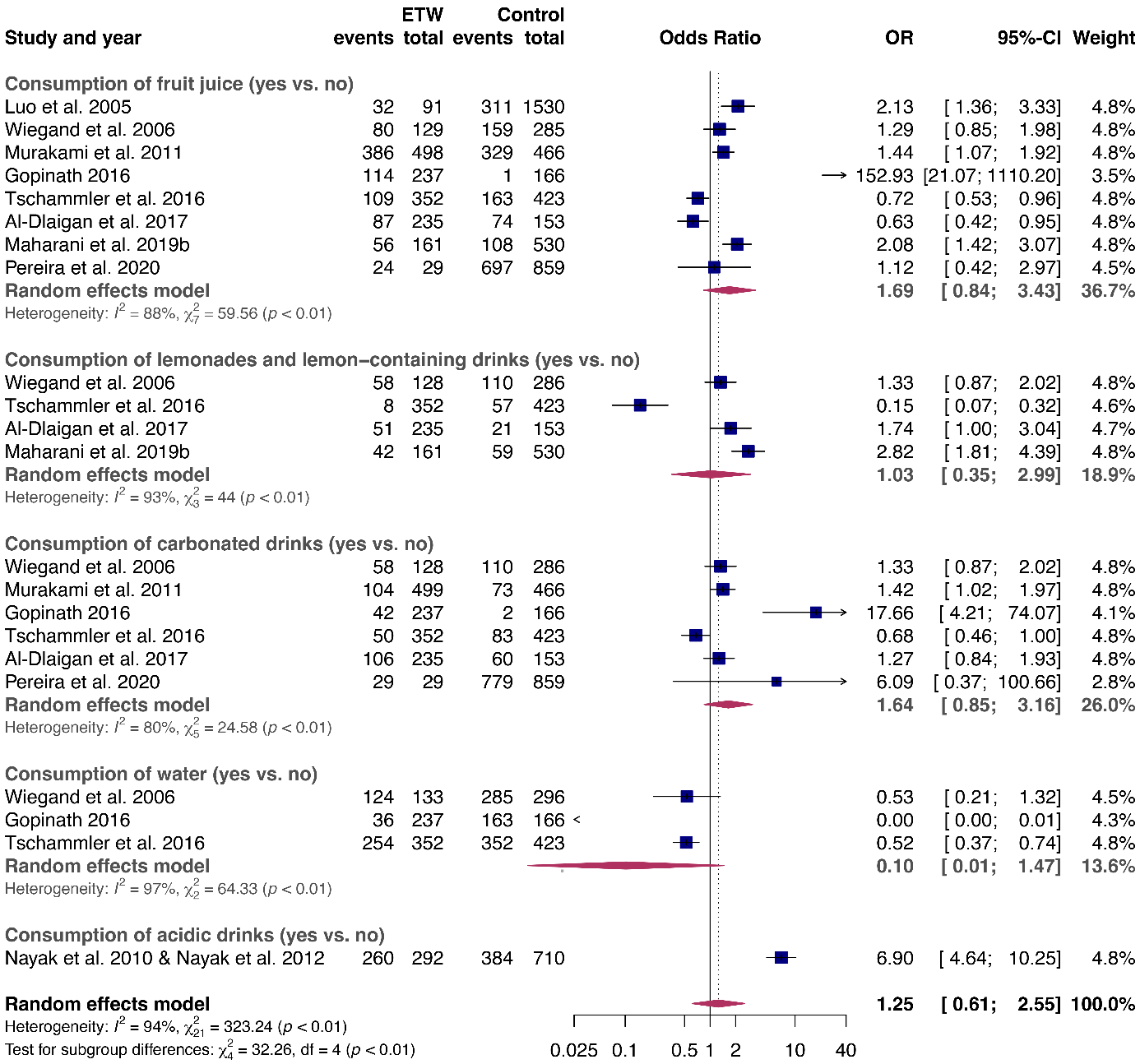


Supplemental Figure 9. Forest plot showing the association of potential risk factors related to beverage consumption and the presence of erosive tooth wear in the primary dentition up to 7 years of age. Odds ratios, 95% confidence intervals, and pooled random-effect estimates (diamonds) are shown. ETW, erosive tooth wear; OR, odds ratio; CI, confidence interval.


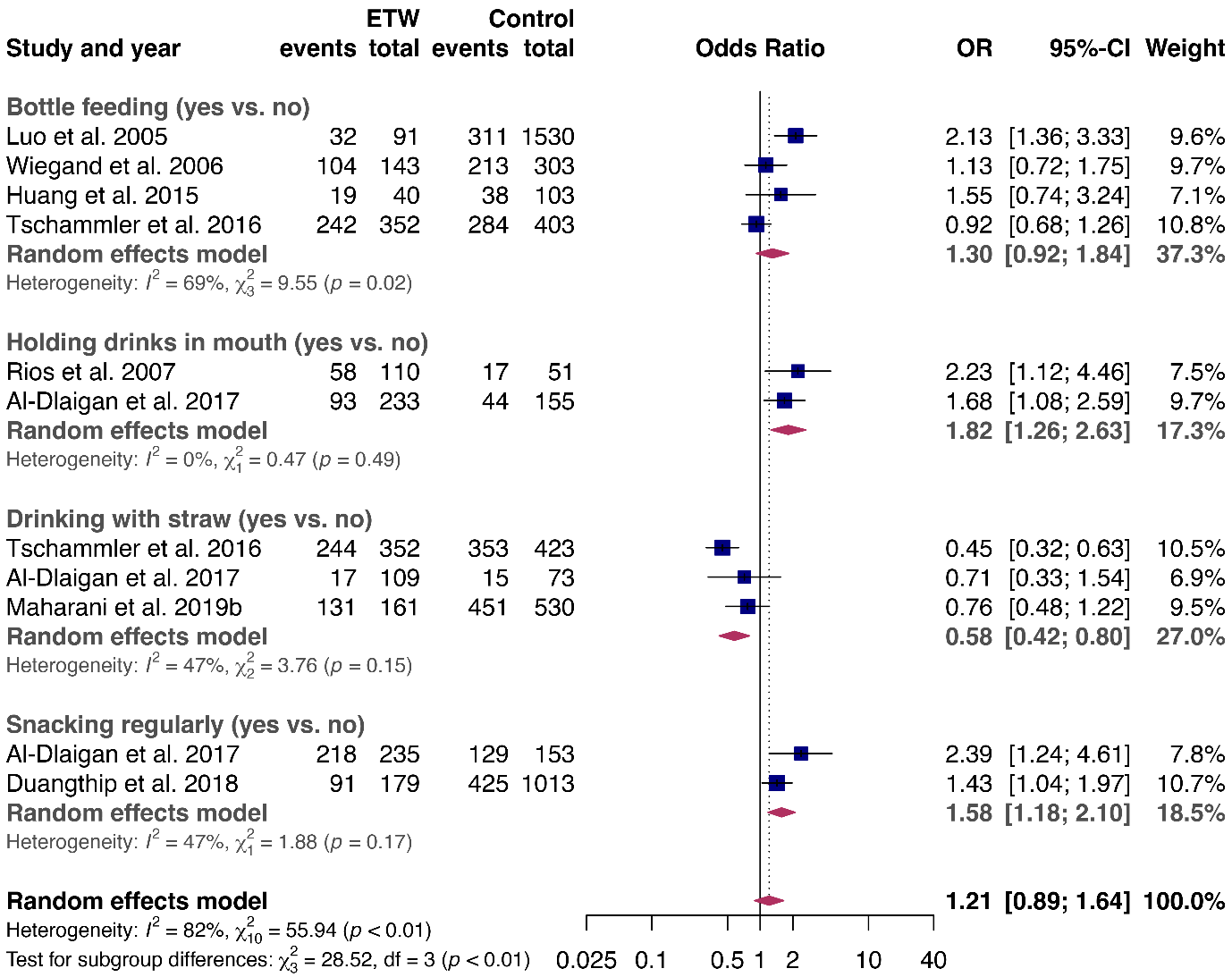


Supplemental Figure 10. Forest plot showing the association of potential risk factors related to dietary habits and the presence of erosive tooth wear in the primary dentition up to 7 years of age. Odds ratios, 95% confidence intervals, and pooled random-effect estimates (diamonds) are shown. ETW, erosive tooth wear; OR, odds ratio; CI, confidence interval.


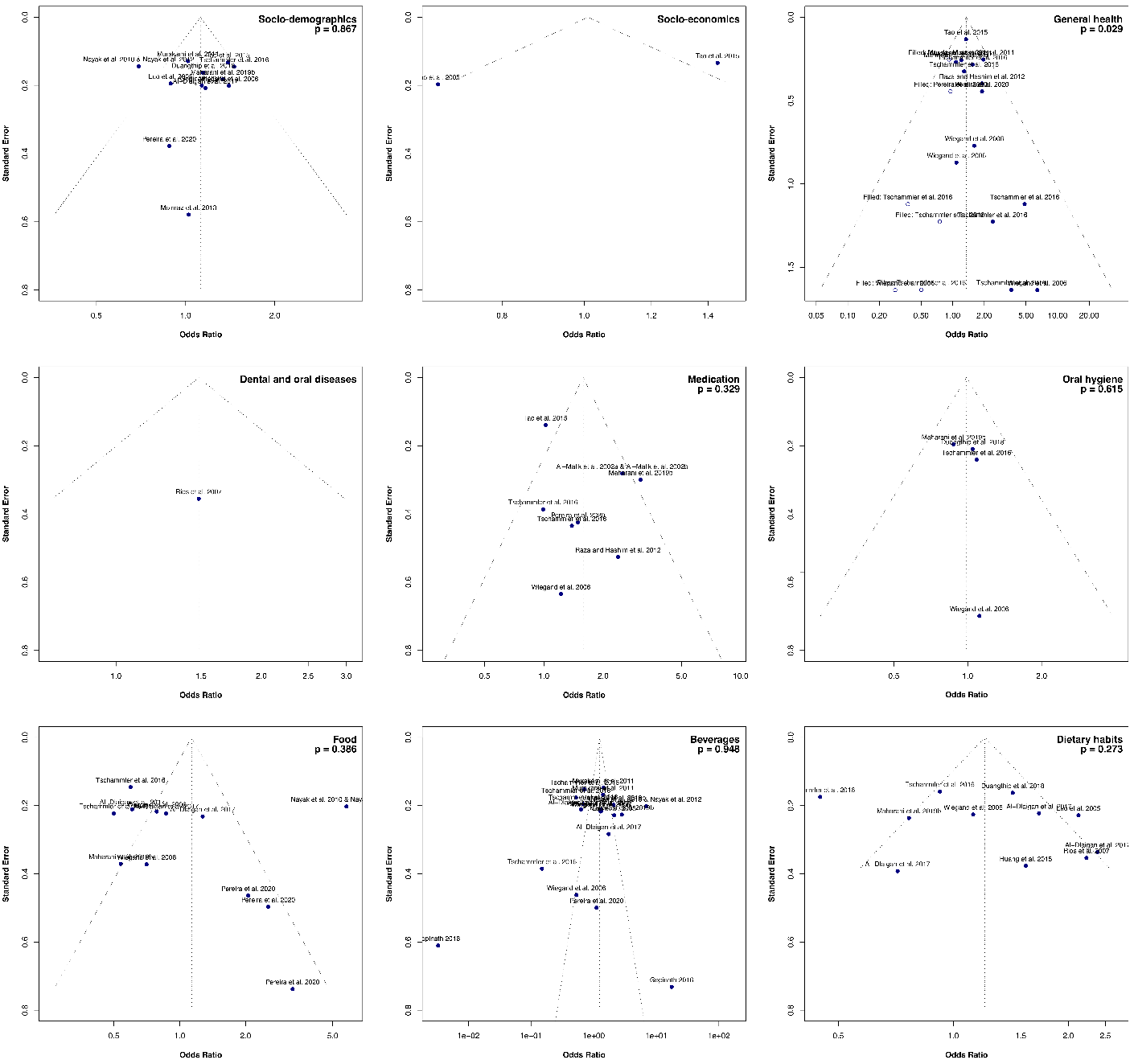
Supplemental Figure 11. Funnel plots for risk factor domains plotting standard errors against the logit of erosive tooth wear prevalence (logit %). Results of Egger’s regression intercept test are shown for domains with ≥3 risk factors. In case of statistically significant asymmetry (p<0.05), imputed missing studies are represented by white circles.
